# A Comparative Analysis to find out the Value of Magnetic Resonance SWI in the Diagnosis of Intracranial Micro-hemorrhage in Patients with Acute Hepatic Encephalopathy

**DOI:** 10.4314/ahs.v25i3.12

**Published:** 2025-09

**Authors:** Guo Dong Pan, Xue Mei Wang, Yao Zhang

**Affiliations:** Department of medical Ultrasound, Beijing Ditan Hospital, Capital Medical University, 100015, Beijing, China

**Keywords:** Magnetic resonance SWI, Acute hepatic encephalopathy, Intra-cranial micro-hemorrhage, Diagnostic value

## Abstract

**Introduction:**

Acute hepatic encephalopathy significantly damages the normal functioning of human nervous system in a very short period of time. AHE gives birth to micro-hemorrhages which increases the morbidity and mortality associated with this disease. Hence, early diagnosis of this disease and its impacts is a key area of interest.

**Objective:**

To investigate value of magnetic resonance susceptibility weighted imaging (SWI) in the diagnosis of intracranial micro-hemorrhage in patients with acute hepatic encephalopathy (AHE).

**Methods:**

85 AHE patients in our hospital from January 2017 to September 2019 were enrolled and received the CT and MRI examinations. The intracranial micro-hemorrhage and lesions detected by CT and MRI were compared to find out the sensitivity of each modality with special focus on SWI technique.

**Results:**

The detection rate of intracranial micro-hemorrhage in SWI was higher than the detection rate of intracranial micro-hemorrhage observed in T1WI, T2WI and CT (P<0.05). Similarly, the detection rate of lesions in SWI was considerably better than the detection rate of such lesions observed in T1WI, T2WI and CT (P<0.05).

**Conclusion:**

Magnetic resonance SWI has significantly better sensitivity in AHE patients with intracerebral micro-hemorrhage, which has proved to be of high value in clinical diagnosis.

## Introduction

Acute hepatic encephalopathy (AHE) is a central neurological dysfunction syndrome resulting from metabolic disorders by liver failure. There are various doctrines for the pathogenesis of the disease, including the doctrine of ammonia poisoning, the doctrine of pseudo-neurotransmitters, γ-Aminobutyric acid/benzodiazepine complex receptor doctrine, etc.^1^. Most consider ammonia poisoning as the main pathogenesis of AHE, when liver failure occurs, the liver's ability to metabolize ammonia decreases. When there is a portal-systemic shunt, intestinal ammonia goes directly into the body circulation without hepatic metabolism, resulting in elevated blood ammonia, while high ammonia allows glial cells in the brain to rapidly convert blood ammonia into glutamine and consume large amounts of adenosine tri-phosphate, making the brain cells energy supply insufficient. While increased intracellular glutamine concentration can increase intracellular pressure and eventually cellular edema occurs^2^. The traditional view was that blood ammonia in the brain acts to elevate osmotic pressure, promote cell swelling, and inhibit oxidation. However, it has been documented that blood ammonia has some effect on the expression of multiple genes, signal transduction pathways, post transcriptional protein modification, which leads to impaired cell function and abnormal cell proliferation until apoptosis^3^. However, the early AHE patients showed no intracranial pressor manifestations, and the symptoms such as vomiting, headache, cognitive decline, and somnolence were the main manifestations. When patients do not accurately express their symptomatic manifestations, it can easily lead to reduce their diagnostic accuracy. When patients present with obvious symptoms, such as liver odor and jaundice, the diagnosis is made, at which point the condition worsens and the timing of treatment is missed^4^. AHE has an acute onset, and the early stage is extremely short, with main symptoms of cognitive dysfunction, headache and vomiting. This disease gives birth to a very diverse spectrum of neuropsychiatric abnormalities which are associated with poor outcomes^5^. The disease can directly cause intracranial micro-hemorrhage with a high rate of disability if not treated promptly^6^. Moreover, minimal to moderate level of acute hepatic encephalopathy is left undiagnosed as majority of the symptoms are ignored for their non specific nature. However, these symptoms greatly affect the quality of life of the affected patients^7^. For this reason, many studies have insisted on early diagnosis of intracranial micro-hemorrhage in AHE is of great significance. However, current methods for diagnosing intracranial micro-hemorrhage in AHE patients are difficult to operate^8^. Therefore, it is necessary to find out a criterion with high diagnostic value. With the development of medical technology, imaging is gradually being widely used in clinical disease diagnosis. Some investigations have proved that the clinical diagnosis of AHE is limited by cranial electron computed tomography (CT), magnetic resonance imaging (MRI), and the lesion needs to be judged with the aid of gradient echo^9^. Susceptibility weighted imaging (SWI) is a modified technique of three-dimensional gradient echo of MRI developed in recent years that takes advantage of the local susceptibility of tissues to form MRI. This study will explore the diagnostic value of SWI in patients with AHE by contrast with CT, conventional serial numbers, which are reported as follows.

## Data and Methods

### Clinical data

For this study a total of 85 AHE patients admitted to our hospital from January 2017 to September 2019 were enrolled. There were 37 males and 48 females, aged 2574 years, with a mean age (50.52 ± 24.98) years. All the enrolled patients and their family members were provided with detailed information about the methods of the study and the procedure involved. They were asked to sign informed consent, which were implemented with the approval of the Medical Ethic Committee. The Inclusion criteria of the study was: 1 Met the relevant diagnostic criteria for AHE and the criteria for intracranial micro-hemorrhage in the “New Advances in the Revised Protocol for the Definition, Nomenclature, Diagnosis and Quantification of Hepatic Encephalopathy” of the World Chinese Journal of Digestology (2003)^5^; 2 Did not incorporate communication barriers; 3 No abnormal metallic substances in the body; 4 No communication barriers; 5 Complete clinical data. Exclusion criteria: 1 Pre-existing neurological disease; 2 Combined congenital incontinentiapigmenti; 3 Co-morbid disease with higher sensitivity to X-rays; 4 Combined immunodeficiency or leukemia prone disease; 5 Gestational women.

### Criteria for intracranial micro-hemorrhage

Intracranial occurrence of abnormal low-density punctate signal shadow, in addition to calcification, cavernous angioma, small vessel flow shadow and other imaging findings, and punctate signal shadow < 5 mm, in a round or ovoid shape with a clear boundary, can be judged intracranial micro-hemorrhage^10^.

### Detection methods

For CT examination the standard procedure were followed which are described as follows: Intravenous iopromide injection^11^ (Xian ling (Guangzhou) Pharmaceutical Co., Ltd., SFDA Approval No. H10970417)2ml/kg) was injected into the patient's elbow 15 min before the examination at a rate of 4 ml per second on a Philips 16 row CT machine, the patient was assisted to take the supine position, setting 200-250 mA current, 8 cm slice thickness and slice distance, setting 120 kV voltage, 512 × 512 matrix, 1.375:1 pitch, scan range: 6 cm from supra-diaphragmatic to infra-diaphragmatic, scan interval: 20s, 50s, thin-section reconstruction was performed to scan arterial phase and venous phase, and finally, the data were transferred to workstation. MRI was performed using a 1.5 T superconducting Siemens MRI scanner with conventional scanner serial numbers: T1WI (TR1750ms, TE25ms), T2WI (TR3275ms, TE107msS) and field of view 240 mm × 240 mm, scan layer spacing 0.5, thickness 5 mm, excitation 1 time, repetition time 25 ms, echo time 20 ms. SWI: TI21ms, TR26ms, scan layer thickness 2 mm, 255 × 158 matrix, excitation 1 tim, 15° deflection angle, 250 kHz common line. All images were discussed and analyzed by 2 experienced radiologists for CT, T1WI, T2WI, SWI images, and the results were agreed on. Focal calcifications and venular opacities were excluded by counting punctate hypo-intense lesions in the cortex, subcortical, brainstem, cerebellum, and thalamus on all of the above sequential imaging maps, and intracranial micro-hemorrhage lesion < 5 mm in diameter on imaging maps were considered positive.

### Statistical methods

Statistical analysis was performed with SPSS 19.0, and chi square (χ2) or Fisher's exact probability method test were used for counting data comparison; all tests determined the P value for inferences, and P < 0.05 indicated a statistically significant difference.

## Results

### General conditions

T1WI in 85 AHE patients revealed 68 ICH patients with 69 lesions, of which 18 had periventricular white matter lesions, 14 had subcortical white matter lesions, 15 had cortical gray matter lesions, 6 had insular cortex lesions, 5 had brainstem lesions, 4 had thalamus lesions, 4 had cerebellum lesions, and 3 splenium of the corpus callosum lesions; T2WI revealed 73 ICH patients with 74 lesions, of which 20 had periventricular white matter, 15 had subcortical white matter, 16 had cortical gray matter, 6 had insular cortex, 5 had brainstem, 4 had thalamus, 4 had cerebellum, 3 had splenium of the corpus callosum, and 1 had a posterior limb of the internal capsule; CT revealed 57 patients with intracerebral hemorrhage with 60 lesions, of which 16 had periventricular white matter, 13 had subcortical white matter, 14 had cortical gray matter, 5 had insular cortex, 4 had brainstem, 3 had thalamus, 3 had cerebellum, and 2 had splenium of the corpus callosum; SWI revealed 85 individuals with ICH and 97 lesions, of which 25 had periventricular white matter, 21 had subcortical white matter, 20 had cortical gray matter, 8 had insular cortex, 7 had brainstem, 6 had thalamus, 5 had cerebellum, 4 had splenium of the corpus callosum, and 1 had the posterior limb of the internal capsule.

### Comparison of detection rates of intracranial micro-hemorrhage by different examination methods

Intracranial micro-hemorrhage was detected more frequently on SWI sequences than on T1WI, T2WI sequences and CT (P < 0.05, Table 1).

**Table 1:** Comparison of detection rates of intracranial micro-hemorrhage by different examination methods

Grouping	Detected	Undetected	Detection rate
SWI(n=85)	85	0	100.00
T1WI(n=85)	68	17	80.00^a^
T2WI(n=85)	73	12	85.88^a^
CT(n=85)	57	28	67.06^a^

**Note:**

a P < 0.05 vs SWI sequence

### Comparison of lesion detection rates by different examination methods

The lesion detection rate of SWI sequences was higher than that of T1WI, T2WI sequences and CT examinations (P < 0.05), see Table 2.

**Table 2:** Comparison of lesion detection rates by different examination methods

Grouping	Number of micro-hemorrhage	Detected	Undetected	Detection rate
SWI	85	97	0	100.00
T1WI	68	69	28	71.13^a^
T2WI	73	74	23	76.29^a^
CT	57	60	37	61.86^a^

**Note:**

a P < 0.05 vs SWI sequence

### MRI and CT clinical findings

**Figure 1:**
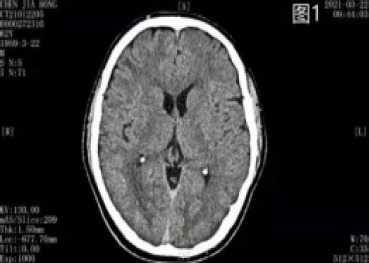
CT showed no obvious abnormality

As shown in Figures 1 ∼ 5

**Figure 2:**
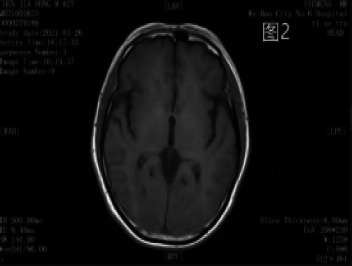
A little punctate hypo-intensity is seen in the basal ganglia bilaterally on T1WI

**Figure 3:**
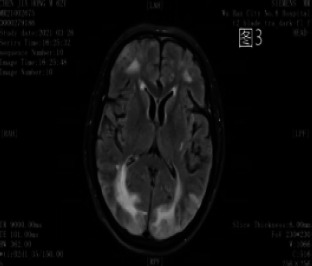
Multiple punctate and patchy hyper-intensities in the left basal ganglia and bilateral frontal and parietal lobes on FLAIR

**Figure 4:**
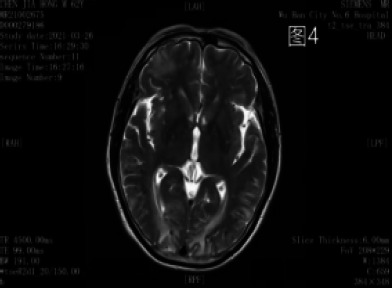
Multiple punctate and patchy hyper/hypo-intensities are seen in the basal ganglia and fronto-occipital lobes bilaterally on T2WI

**Figure 5:**
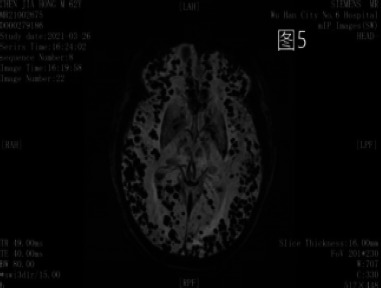
Multiple and patchy hypo-intensities were seen in bilateral basal ganglia and bilateral cerebral hemispheres by SWI

## Discussion

Acute hepatic encephalopathy rapidly damages the nervous tissues with significant level of morbidity^12^. For this reason, early diagnosis of AHE is of great significance.

With the development of medical technology, imaging examination becomes an important means to examine diseases. Currently, MRI and CT imaging are mostly used to diagnose brain diseases in clinic^13^. SWI is an imaging modality in the development of MRI with the advantages of thin-layer thick, multidirectional complete flow compensation, which effectively avoids signal loss while reducing the adverse effects of phase mapping, and this technology utilizes phase masks for image enhancement processing. The unique image processing and data acquisition modality of SWI can greatly improve the quality of image contrast and detect intracranial micro-hemorrhage lesions more sensitively than non-enhanced CT. Intracranial micro-hemorrhage is lesions of intracranial blood tubules that cause clinical damage to the brain parenchyma with surrounding hemosiderin deposition. With a large number of studies, intracranial micro-hemorrhage has been found to have a correlation with cognitive dysfunction in patients^14^. However, the causes of cognitive impairment in patients may be intracranial vasculo-pathy, hemorrhage area size, volume, and so on. Another study noted that cognitive domain impairment was correlated with basal nuclei, frontal lobe in patients with intracranial micro-hemorrhage^15^. Intracranial micro-hemorrhage can also cause central choline transmitter delivery to be affected, which in turn can affect patient orientation, concentration, and other symptoms. It has been reported in the literature that intracranial micro-hemorrhage is a hallmark of intracranial small vessel disease and can be associated with intracerebral hemorrhage, ischemic stroke, etc., and that patients' recurrent intracranial hemorrhage is positively correlated with patients' age and intracranial hemorrhage lesions^16^. It follows that when a patient has an increased number of lesions of intracranial hemorrhage, the patient has an increased risk of intracranial hemorrhage, which can be directly fatal in severe cases. On MRI conventional sequence scans, it is seen that T1WI and T2WI sequences correlate with cerebral white matter signal in cerebral edema and related cerebral tubulo-pathies in patients with AHE^17^. The abnormalities exhibited by each sequence are reversible and occur with specific locations. There are reports in the literature that patients with chronic hepatic encephalopathy were detected by MRI imaging for with lesions, and hyper-intensity of the globus pallidus can be seen on T1WI. But this conventional MRI sequence has low accuracy to reflect the situation of intracranial hemorrhage lesions in AHE patients^18^. The results of this study show that the detection rate of intracranial micro-hemorrhage is higher on SWI sequences than on T1WI, T2WI sequences and CT. The lesion detection rate of SWI sequence was higher than that of T1WI, T2WI sequence and CT examination. It is suggested that SWI has a significant sensitivity for the manifestation of intracranial micro-hemorrhage lesions, which can be beneficial for differentiating the intracranial hemorrhage lesions that are not demonstrable on conventional sequence scans from other lesions^19^. In conclusion, magnetic resonance SWI has significant sensitivity in AHE patients with intracranial micro-hemorrhage and has high value in clinical diagnosis, providing effective value in clinical settings^20^. This study has played a significant role in manifesting the supremacy of MRI in detection of intracranial hemorrhages in patients suffering from AHE. However, this study also has some limitations. One such limitation is limited sample of the study and the short duration of research. Keeping in view the clinical significance of the issue highlighted by this study further inquiry on this topic is the need of the hour.
